# Glasgow Royal Infirmary

**Published:** 1831-08

**Authors:** 


### HOSPITAL REPORTS.

### GLASGOW ROYAL INFIRMARY.

*Cases of Aneurism.* By Dr. Perry, one of the Surgeons to the Glasgow Royal Infirmary.*

The following cases came under my care, while surgeon in the Glasgow Royal Infirmary, in 1820. The first case is chiefly remarkable for the secondary hemorrhage which occurred several weeks after the operation of tying the artery, and the manner the hemorrhage was suppressed. Unless it had taken place in a public hospital, reasonable doubts might have existed that there was some deception; but the nature of the hemorrhage was fully ascertained, by a number who witnessed it, and when Dr. Wallace, the house surgeon, was summoned from his apartment, he purposely removed the pressure, and allowed the blood to flow, that he might fully ascertain its character. From the size of the tumor, and the small space left betwixt it and the clavicle, the artery must have been tied very near its separation from the subclavian, and the introduction of the probe may have disturbed the clot formed, while the adhesion of the sides of the artery had not fully taken place.

This patient, M'Arthur, called at the hospital at the visiting hour, in the month of February last, for the purpose of receiving a certificate of his situation, as he had been called, along with the other pensioners, to appear before the district surgeon, that his

* Glasgow Med. Journal.

fitness to serve his Majesty might be ascertained. In the site of the aneurismal tumor there is now a hollow, and the skin firmly adherent to the upper and lateral portion of the thyroid cartilage; the slightest pressure upon the part excites a sudden feeling of suffocation and coughing. The pulsation of the temporal and labial arteries cannot be distinctly felt. In other respects in good health.

The second case of aneurism of the femoral artery is only remarkable for its situation, and the alleged cause of its origin. It appears that the sudden impulse given to the blood in the artery had produced partial rupture of its coats, which afterwards gradually yielded, forming the aneurismal tumor.

14th Nov. 1820. Peter M'Arthur, æt. thirty-nine. On the right side of the neck, in the course of the carotid artery, there is a pretty firm, ovoid, pulsating, painful tumor, which occupies nearly the upper two thirds of the neck. The account he gives of its origin and history is rather indistinct. It appears, that upwards of two years ago, a small painful swelling, about the size of a field bean, formed behind the angle of the jaw. This gradually got larger, and having, in the course of about eight weeks, attained nearly the magnitude of the fist, and being at that time soft on its summit, it was accordingly punctured, when scarcely two drachms of purulent fluid, mixed with blood, issued. After this, part of the tumor fell into an imperfect state of suppuration, and finally, the puncture degenerated into a very extensive sore; since then, this has continued to discharge matter, although frequently, during this period, it has been very nearly healed up. He first observed the pulsating tumor described, rather more than a year ago. It may, he says, have existed previous to that time, as his attention was only directed to it after the subsidence of the tumefaction and induration surrounding the ulcer. This tumor, which at first was very small, and situated at the upper part of the sore, has grown gradually larger, although with much more rapidity within the last nine weeks than previously: at that time a bursting took place at the part exposed by the sore, and, before it could be checked, (which was effected by pressure on the course of the artery above the hollow of the sternum,) he lost a considerable quantity of blood. The same thing has taken place five times at irregular periods since; the last time at three o'clock this morning, when he is said to have lost fourteen ounces of blood, a quantity considerably greater than at any former period. By the surgeon's report who saw him at that time, the blood was distinctly arterial. The ulcer mentioned, which is about the diameter of a shilling, is occupied by a firm coagulum. Is of a strumous habit.

Three years ago had primary syphilis, and a year after ulcerated throat, both of which were cured by the use of mercury. About fourteen weeks since was seized with severe headach, which was considered rheumatic, and treated accordingly, but without the least relief. It became immediately easier after the first attack of hemorrhage, and he has had no remains of it since the second attack. Flesh and strength much reduced, in consequence of close confinement to bed during the last fourteen weeks.

Was brought to this hospital at three o'clock this afternoon, when a consultation having immediately met, it was resolved not to hazard the operation till some change for the better should take place on his pulse, which at that time could with difficulty be numbered at the wrist. His extremities were also cold, and he had a feeling of slight depression about the præcordia. Warm cloths were, accordingly, ordered to his feet, and he was to have some beef-tea, or any other nutritious diet. The consultation again met at six o'clock. The pulse at that time was upwards of 150, and in other respects very little improved; the operation was recommended to be delayed till the morning. In the mean time the patient to be strictly watched, so that, on the accession of the least hemorrhage, the operation might be had recourse to immediately. This, accordingly, took place at eleven o'clock P.M., and in an hour after the operation was done in the following manner: An incision two inches and a half long was made along the inner edge of the sterno-mastoid muscle, extending from a little above the lower part of the tumor to the sternum, whence another incision about half the extent was carried along the upper margin of the clavicle. The flaps thus made were then reflected, and the sterno-hyoid and mastoid muscles being held apart, the sheath of the vessels were, after a short time, completely brought into view. This stage of the operation was effected by the finger and handle of the scalpel, and but little interruption was occasioned by the internal jugular. The sheath was then opened in a longitudinal direction, and the artery being separated all round, a single ligature was conveyed under it, from within outwards, and tied, when all pulsation in the tumor instantly ceased. The edges of the wound were then brought together and retained by straps. The operation lasted nearly half an hour, and during that time scarcely a teaspoonful of blood was lost. The operation was frequently interrupted by the cough and occasional retching. At present (ten o'clock A.M.) complains of no uneasiness except slight cough, and has remained in a quiet state since the operation. Pulse about 140, firmer. Required a little brandy and water occasionally.*

16th. Would have rested well, but for the slight tickling cough; expectoration suppressed since the operation. Experiences a trifling degree of cold in the right side of the neck, but there is no change of temperature discoverable by the touch; pulse 120, firmer. Has taken occasionally a little arrowroot. One stool

* The narrative of the case, consultation, and subsequent operation, was drawn up by my friend Dr. Auchincloss, who at that time ably filled the situation of surgeon's clerk; during the operation I was assisted by my colleague, the late Dr. G. C. Monteath.

from an enema. Some of the dressings which were loosened, being removed, the under part of the wound seems united. Habt. Mist. mucilag.

17th. Pulse 110, firm. The coagulum has come away from the ulcer, on anterior part of aneurismal tumor, which is subsiding rapidly. The ulcer clean. No stool since enema. Hab. stat. Infus. Sennæ ℥iv. c. Sulph. Magnes. ℥ss.

18th. Felt last night a little giddy, but this went off by the morning; rested well; pulse 100; as yet no stool, but feels as if medicine would operate soon.

19th. Free alvine evacuation; pulse 104. Tumor more than a half less since the operation.

20th. Complains of pain about the thyroid cartilage when he coughs or swallows, and of a feeling of uneasiness in right side of head. Bowels kept open by mild laxatives. Pulse 108.

24th. Pain in site of the ulcer, where there is some hardness. Appetite good; pulse 100, soft. Sleep refreshing. Adhib. ulcer. cataplasm.

27th. Pain about the ulcer nearly gone. Ligature came away this morning. Cough easier. Bowels regular.

Dec. 3d. Pain and hardness in site of aneurismal tumor, and pain going up the side of head. Cough troublesome. Cont. catap. et Mist. mucilag.

5th. Yesterday a discharge of matter from the ulcer. Pain in site of aneurismal tumor and right side of head abated. Fungous granulations have arisen from the wound. To be touched with Sulph. Cupri.

11th. Little discharge from the ulcer. Tumor gone, but its site feels indurated. Says he feels better than he has done for the last twelve months. A small compress to be applied to the fungous granulations from the site of the incision.

29th December. Since last report has enjoyed good health, though weak, with the exception of the cough being at times a little troublesome. A small quantity of purulent matter continued to be discharged from the site of the aneurismal tumor till within these few days. The wound by the incision healed up about ten days ago, with the exception of a little redness and serous oozing from the spot where the ligature was. He began to complain of some pain on coughing, and a sensation of throbbing in the part, which was succeeded by suppuration, and purulent matter continued to be discharged for three or four days. Fungous granulations formed similar to what takes place when there is a portion of diseased bone underneath, and were repeatedly touched by escharotics. On introducing the probe into the wound today, it passed fully an inch backwards, and could be moved freely around, as if there was a cavity. The aneurismal tumor completely gone, and the ulcer, which has been open for two years, quite healed.

30th. Last night, at six o'clock, while throwing off his coat to go to bed, he was alarmed by feeling blood running profusely down his breast. He immediately applied his finger to the spot, and stopped it; when the finger was removed, blood of a florid colour issued, *per saltum*, from the wound, and to Dr. Wallace,* the house surgeon, who was hastily summoned and saw it, the stream appeared equal to that from a large temporal artery when opened. Pressure was continued for about an hour, when a coagulum had formed, and no more blood came, till eight o'clock. A little bleeding occurred at that time, but was easily repressed by the finger and a graduated compress, and a bandage being then applied, the bleeding was stopped till four o'clock next morning. At that hour, while lying in bed after a painful sensation of throbbing in the wound, a gush of blood burst forth, making its way through the folds of the bandage, which were instantly cut and the finger applied, but it was not so easily stopped as formerly; the current continued for some time. It was represented by the nurse as rising several inches high, in a full jetting stream, as large as that coming from the arm in phlebotomy. The bleeding was stopped, and a coagulum formed, and it has not since returned. The quantity of blood lost could not be accurately ascertained, but might be, the last time, from twelve to sixteen ounces. Had a rigor soon after the first bleeding, after which the pulse rose to 104, and became sharper. A consultation being called on the case today, it was gravely discussed whether the arteria innominata might not be tied. It was agreed that the patient should be kept quiet, upon the lowest diet, and that a firm bandage should be applied. A compress bandage and a herniary truss were firmly applied with a torniquet, brought over the right shoulder and under the left arm, the pad above the wound. Eight o'clock, P.M. pulse 100, soft. Has taken nothing since yesterday but water gruel.

31st. No return of bleeding. This morning, about four o'clock, had the painful sensation which he says formerly preceded the flow of blood; bandage firm; pulse ninety-six, soft; tongue clean; mind considerably agitated. To have a colocynth pill at bedtime.

2d January, 1821. No return of hemorrhage; pulse down to eighty-two, soft. The bandage being removed today, the wound appeared closed. No pulsation felt. The bandage was again firmly applied; mind composed.

12th. Diet has been very spare. Bandage kept firmly applied. No return of hemorrhage. There is still a small fungus around the spot where the ligature was. The skin around this has been abraded by the compress. Complains of a feeling of numbness in that side of the neck, particularly around site of the former ulcer. There is a light puffy swelling at the angle of the jaw. General health continues good. Bandage to be lightly applied. Pulse natural; tongue clean. Better diet.

24th. Since last report the fungus was occasionally touched

* Dr. Wallace succeeded Dr. Auchincloss as surgeon's clerk, on 1st December, 1820.

with sulphas cupri, and then dressed with simple ointment; it has now been skinned over for a week. The swelling at the angle of the jaw has disappeared, as well as the feeling of the numbness lately complained of. The temporal artery of the right side is felt beating distinctly, but weak. He enjoys better health at present than for many years past. Dismissed cured.

James King, butcher, æt. forty-five. January 1821. About five inches below Poupart's ligament, on the outside of the right thigh, there is a pulsating tumor about four inches in diameter. It is extremely tense, but may be greatly diminished in size by firm pressure continued for some time, though it resumes its former bulk the instant the hand is removed. This swelling was first noticed a month ago, and was then the size of a small plum, and slightly painful: at the end of three weeks it had attained the size of an hen's egg, and began to produce lameness and pain of the limb below. The pulsation was also first noticed about this time. Pulse 100, rather weak. Tongue white in the middle. General health good. An inch and a half below the inferior margin of the swelling, (i.e. ten inches below Poupart's ligament,) but a little dextrad of the artery, the skin is slightly discoloured, in consequence, he says, of a kick from an ox six weeks before the tumor was discovered. The pain occasioned by this injury soon wore off, and he continued his daily avocations as usual.

19th. A consultation being held on the case, it was agreed that the artery should be cut down upon, which was done on the 20th, and tied immediately above the tumor with a single ligature. Considerable hemorrhage occurred during the operation, and two arteries besides the femoral were tied, but the ligatures came away immediately afterwards, and the bleeding did not recur.

23d. No particular change has been observed in the heat of the limb, but a slight lividity was noticed in the right foot the day after the operation. The uneasiness complained of in the limb previous to the operation has gradually subsided. Pulse down to seventy; tongue cleaner. The aneurismal tumor sensibly decreased in size. A large quantity of pus is discharged from the wound.

28th. After last report, the discharge of pus became more profuse, wetting the bandages soon after their application. Compresses have been applied to both sides of the wound, and the quantity of pus is much diminished.

February 10th. Wound healed. Tumor diminished to about a third of its former size. General health good. Can walk freely. Dismissed cured.

